# Sepsis Caused by Ewingella americana in an Immunocompromised Patient: A Case Report

**DOI:** 10.7759/cureus.85641

**Published:** 2025-06-09

**Authors:** Hala Jafarova Ayik, Cagla Eyupler, Gizem Yassa, Cagri Aksu, Nurcan Duman

**Affiliations:** 1 Internal Medicine, Marmara University School of Medicine, Istanbul, TUR; 2 Internal Medicine, Göztepe Prof. Dr. Süleyman Yalçın City Hospital, Istanbul Medeniyet University, Istanbul, TUR; 3 Internal Medicine, Northwestern Medicine McHenry Hospital, McHenry, USA; 4 Internal Medicine, Tower Health Phoenixville Hospital, Phoenixville, USA; 5 Microbiology, Marmara University School of Medicine, Istanbul, TUR

**Keywords:** cancer patient, chemotherapy, ewingella americana, immunocompromised patient, red blood cell transfusion, sepsis

## Abstract

Human infections caused by *Ewingella americana* have been rarely described. We report a case of *E. americana* in a cancer patient undergoing chemotherapy, who developed sepsis during a red blood cell transfusion. This causative pathogen was identified through the patient’s blood culture. The patient was treated with ceftriaxone and showed complete clinical improvement, with no further growth of *E. americana* or other pathogenic isolates in subsequent blood cultures. To the best of our knowledge, this is the first reported case of *E. americana* infection in a cancer patient undergoing chemotherapy in Türkiye.

## Introduction

*Ewingella americana *is a gram-negative, lactose-fermenting, oxidase-negative, indole-negative, catalase-positive, facultative anaerobic bacillus that was first identified by Grimont et al. in 1983 [1]. It belongs to the order *Enterobacterales* and the family *Yersiniaceae* and is the only species within its genus. This organism is unusual and has rarely been reported as an opportunistic human pathogen. The source of *E. americana* and its natural habitat are poorly understood. Some investigations have revealed its presence in conjunctiva [2], peritoneal dialysate [3,4], wound [5], synovial fluid [6], sputum [7], and heart and spleen blood from a fatal case of Waterhouse-Friderichsen syndrome [8]. However, the most common human source identified so far has been blood [9,10].

Although there are some reports of this infection in previously healthy individuals [2,8], this pathogen mostly infects immunocompromised patients, such as those with diabetes, renal failure, drug abuse, and chemotherapy. There are case reports of *E. americana* causing osteomyelitis and septic arthritis in an intravenous drug abuse patient [6], catheter-related bacteraemia in a chemotherapy patient [9], and nosocomial bacteraemia in intensive care unit patients who had undergone cardiovascular or peripheral vascular surgery [10]. There is a case report of an outbreak of *E. americana* pseudobacteraemia, most likely resulting from cross-contamination of blood culture bottles, where nonsterile coagulation tubes contaminated with *E. americana* were filled first [11].

Although human infections caused by this pathogen are rare, this microorganism can lead to significant clinical consequences, especially in immunosuppressed patients when they occur. In this case report, we describe the isolation of *E. americana* from blood cultures of an immunocompromised patient who developed sepsis during erythrocyte suspension transfusion.

## Case presentation

A 21-year-old male patient with no significant medical history presented with abdominal pain and dark stool. On examination, he was found to be anaemic. An upper gastrointestinal endoscopy and biopsy were performed for further evaluation, which revealed choriocarcinoma. Following a thorough diagnostic imaging workup, a retroperitoneal mass and testicular lesion were identified. As a result, the patient was diagnosed with testicular choriocarcinoma with retroperitoneal metastasis and duodenal invasion.

He underwent a right radical inguinal orchiectomy, followed by the initiation of BEP (bleomycin, etoposide, and cisplatin) chemotherapy. On the second day of BEP chemotherapy, an erythrocyte suspension transfusion was planned due to the patient's haemoglobin level of 6.7 g/dL, accompanied by fatigue. During the transfusion, the patient became unwell and showed symptoms of nausea, vomiting, chills, headache, and shortness of breath. Notably, this was not his first erythrocyte suspension transfusion; he had previously undergone several uncomplicated transfusions due to a history of gastrointestinal bleeding and haemoglobin levels below 7 g/dL.

On examination, his GCS (Glasgow Coma Scale) was 15. He was tachypnoeic, hypoxic on room air, and hypotensive (Table 1). The rest of the examination was unremarkable. Subsequent chest radiography revealed normal findings. Initially, it was thought to be a transfusion-related anaphylactic reaction, and intramuscular adrenaline was administered. The patient was managed in accordance with the anaphylaxis guidelines.

**Table 1:** Vital signs on assessment MAP: mean arterial pressure; qSOFA: Quick Sequential Organ Failure Assessment; NEWS2: National Early Warning Score 2

Parameters	Patient Values	Reference Ranges
Blood pressure	67/34 mmHg	90/60-120/80 mmHg
MAP	45 mmHg	70-100 mmHg
Pulse rate	98 beats per minute	60-100 beats per minute
Respiratory rate	24 breaths per minute	12-20 breaths per minute
Oxygen saturation	87% on room air	≥96%
Initial temperature	36.6 °C	36.5-37.2 °C
Temperature in the following 30 minutes	≥39.1 °C	36.5-37.2 °C
qSOFA	2	0 to 1 point not high risk; 2 to 3 points high risk
NEWS2	11	0 to 4 points low risk; 5 to 6 points medium risk; 7 or more points high risk

Despite optimal management, the patient remained tachypnoeic and hypotensive, and developed a high fever of 39.6°C, which persisted for the following 30 minutes. His Quick Sequential Organ Failure Assessment (qSOFA) and National Early Warning Score 2 (NEWS2) scores indicated a high risk of early sepsis (Table 1). As a result, he was started on piperacillin-tazobactam and vancomycin treatment based on local guidelines after one set of blood cultures was taken. Laboratory findings were also consistent with infection, showing neutrophilic leukocytosis, elevated C-reactive protein, markedly raised procalcitonin levels, and increased lactate levels (Table 2).

**Table 2:** Laboratory findings BUN: blood urea nitrogen; CRP: C-reactive protein; ABG: arterial blood gas; PaCO2: partial pressure of carbon dioxide; PaO2: partial pressure of oxygen

Parameters	Patient Values	Reference Ranges
White blood cells	18.2 x 10^3^/μL	4-10 x 10^3^/μL
Neutrophils %	98.1	37-73
Haemoglobin	6.1 g/dL	12.0-17.0 g/dL
Platelets	350x10³/μL	150-440×10³/µL
BUN	9 mg/dL	6-23 mg/dL
Creatinine	0.97 mg/dL	0-1.2 mg/dL
Albumin	2.3 g/dL	3.5-5.4 g/dL
CRP	92.70 mg/L	0-5 mg/L
Procalcitonin	40.73 ng/mL	0-0.5 ng/mL
ABG lactate level	5.7 mmoL/L	0.5-1.6 mmol/L
pH	7.39	7.35-7.45
PaCO2	30 mmHg	35-48 mmHg
PaO2	70 mmHg	83-108 mmHg

Aerobic and anaerobic blood culture bottles were incubated in the BD BACTEC™ FX Blood Culture System (Becton, Dickinson and Company, Franklin Lakes, New Jersey, United States). The aerobic bottle signalled positive after 19 hours of incubation. Gram stain from the positive vial revealed gram-negative bacilli. Subcultures showed non-lactose fermenting colonies on MacConkey agar and large yellowish colonies on chocolate agar after 24 hours of incubation at 37°C. The colonies were oxidase-negative and catalase-positive.

The isolate could not be identified by VITEK® MS (bioMérieux SA, Marcy-l'Étoile, France). For identification, VITEK 2 GN (bioMérieux SA) was used and identified as *E. americana* with 99% confidence. For antimicrobial susceptibility testing, VITEK 2 AST-N325 (bioMérieux SA) was used. Antimicrobial susceptibility results of the isolate are listed in Table 3. The second blood culture set also revealed the growth of the same microorganism.

**Table 3:** Antimicrobial susceptibility results of Ewingella americana

Antimicrobial agents	Interpretation
Ampicillin	Resistant
Amoxicillin-clavulanate	Resistant
Cefazolin	Resistant
Cefuroxime	Resistant
Cefuroxime axetil	Resistant
Cefoxitin	Resistant
Ceftazidime	Sensitive
Ceftriaxone	Sensitive
Cefepime	Sensitive
Piperacillin – tazobactam	Sensitive
Meropenem	Sensitive
Gentamicin	Sensitive
Ciprofloxacin	Sensitive
Tigecycline	Sensitive
Trimethoprim-sulfamethoxazole	Sensitive

The therapy was narrowed to ceftriaxone once antibiotic susceptibility results were available. The patient's clinical and laboratory findings subsequently improved, and the antibiotic treatment was completed over two weeks with full recovery.

## Discussion

*E. americana* is a gram-negative rod and has rarely been reported as a cause of human infections. A literature review revealed associations of *E. americana* with conjunctivitis [2], peritonitis [3,4], wound infection [5], osteomyelitis and septic arthritis [6], pneumonia [7], and bacteraemia [9,10]. To the best of our knowledge, this is the first reported case of E. americana infection in a cancer patient undergoing chemotherapy in Türkiye.

In our case, the patient was immunocompromised due to both cancer and chemotherapy treatment, which made him susceptible to *E. americana* infection. However, the exact origin of the infection was not definitively determined. Since a culture was not taken from the transfused blood at the same time, it could not be confirmed whether the transfusion itself was the source. We believe that either the contaminated erythrocyte suspension or the surgical equipment used during the duodenal biopsy and inguinal orchiectomy could have been the possible origins. We mostly speculate that the erythrocyte suspension was the likely source, as the patient developed sepsis during its transfusion.

The erythrocyte suspension may have been contaminated during the preparation phase, considering that *E. americana* grows favourably at 4°C and can exist in water, and citrate solutions prepared in the hospital may act as a nosocomial reservoir [11]. Domestic water [3,4], inadequate hand hygiene [9], contamination of solutions used to flush catheters [9], and contaminated ice bath [10] have also been proposed as other sources.

In our case, the patient became acutely unwell, with respiratory distress and hypotension during an erythrocyte suspension transfusion. We suspected serious transfusion reactions, including anaphylaxis, transfusion-related acute lung injury (TRALI), transfusion-associated circulatory overload (TACO), and sepsis. Although anaphylaxis was initially considered due to the presence of respiratory distress and hypotension, there was no angioedema, wheezing, and the patient did not improve after management with adrenaline. Respiratory distress and hypotension can also occur in TRALI, but the presence of a normal chest examination and radiography makes this diagnosis less likely. The absence of hypertension and positive fluid balance, along with a normal chest examination and radiography, helps exclude TACO [12]. As there was no circulatory overload, no rales on chest examination, and no evidence of pulmonary oedema on chest radiography in our patient, both TRALI and TACO were excluded.

Our definitive diagnosis was sepsis, accompanied by high-grade, persistent fever and elevated qSOFA and NEWS2 scores. It was later confirmed by blood cultures and blood tests. There is a case report of platelet transfusion-related *Serratia marcescens* sepsis, in which an allergic transfusion reaction was initially suspected, but sepsis was later confirmed through the isolation of the pathogen from both the patient’s blood culture and the platelet bag [13]. We believe this report shares common aspects with our case.

In a study conducted by Stock et al., the susceptibility of 20 *E. americana* strains to 72 antibiotics was tested. The results showed that *E. americana* strains were naturally resistant or of intermediate susceptibility to first- and second-generation cephalosporins, benzylpenicillin, oxacillin, fosfomycin, erythromycin, glycopeptides, and rifampicin. Uniform natural sensitivity was found with acylureidopenicillins (except for azlocillin), carbapenems, aminoglycosides, quinolones, and azithromycin. The strains were naturally sensitive or of intermediate susceptibility to aminopenicillins (with and without β-lactamase inhibitors), azlocillin, and nitrofurantoin [14]. In our case, antimicrobial susceptibility of *E. americana* was largely consistent with the results of this study, except for the observed resistance to aminopenicillins.

There is a case report of multidrug-resistant *E. americana*, resistant to all antibiotics except trimethoprim/sulfamethoxazole, ticarcillin/clavulanate, and cefotetan, in a patient with an exacerbation of chronic obstructive pulmonary disease (COPD) [15]. 

Our case is notable because it emphasizes that, although sepsis is a relatively rare reaction, it should still be considered among the differential diagnoses when a patient becomes unwell during a transfusion. It also highlights the importance of *E. americana* as a critical pathogen, especially in immunosuppressed patients, and its multidrug-resistant nature should also not be overlooked.

## Conclusions

Although *E. americana* primarily causes infection in immunosuppressed patients, there have been cases of *E. americana* infection in previously healthy patients as well. Therefore, it remains debatable whether *E. americana* is an opportunistic or true pathogen. Thus, its effect on both groups should be more widely established. Due to its association with life-threatening conditions, especially in immunosuppressed patients, its pathogenic potential and the mechanisms underlying antimicrobial resistance should be investigated in detail. More studies are needed on its ecology and epidemiology to define its clinical significance.
